# AxIOM: Amphipod crustaceans from insular *Posidonia oceanica* seagrass meadows

**DOI:** 10.3897/BDJ.4.e10109

**Published:** 2016-09-08

**Authors:** Loïc N. Michel, Nicolas Sturaro, André Heughebaert, Gilles Lepoint

**Affiliations:** ‡ Laboratory of Oceanology, FOCUS research unit, University of Liege, Liege, Belgium; § Belgian Biodiversity Platform, Bruxelles, Belgium

**Keywords:** Amphipoda, Crustacea, Posidonia oceanica, Seagrass, Mediterranean, Corsica, Sardinia, Islands, Revellata Bay, Tavolara - Punta Coda Cavallo Marine Protected Area, Hierarchical sampling design

## Abstract

**Background:**

The Neptune grass, *Posidonia oceanica* (L.) Delile, 1813, is the most widespread seagrass of the Mediterranean Sea. This foundation species forms large meadows that, through habitat and trophic services, act as biodiversity hotspots. In Neptune grass meadows, amphipod crustaceans are one of the dominant groups of vagile invertebrates, forming an abundant and diverse taxocenosis. They are key ecological components of the complex, pivotal, yet critically endangered Neptune grass ecosystems. Nevertheless, comprehensive qualitative and quantitative data about amphipod fauna found in Mediterranean Neptune grass meadows remain scarce, especially in insular locations.

**New information:**

Here, we provide in-depth metadata about AxIOM, a sample-based dataset published on the GBIF portal. AxIOM is based on an extensive and spatially hierarchized sampling design with multiple years, seasons, day periods, and methods. Samples were taken along the coasts of Calvi Bay (Corsica, France) and of the Tavolara-Punta Coda Cavallo Marine Protected Area (Sardinia, Italy). In total, AxIOM contains 187 samples documenting occurrence (1775 records) and abundance (10720 specimens) of amphipod crustaceans belonging to 72 species spanning 29 families. The dataset is available at http://ipt.biodiversity.be/resource?r=axiom.

## Introduction

The Neptune grass, *Posidonia oceanica* (L.) Delile, 1813, is the most widespread seagrass of the Mediterranean Sea. This foundation species forms large meadows that are of crucial ecological and economic importance. Their complex, multi-layered structure offers a suitable habitat to hundreds of animal and plant species, as well as micro-organisms (Buia et al. 2000). In addition, through the epiphytes that grow on all parts of the plants, its dead and decaying tissues and, to a lesser extent, its living tissues, *P. oceanica* supports elaborate food webs (Vizzini 2009. Thanks to these habitat and trophic services, Neptune grass meadows, which cover up to 50000 km^2^, are biodiversity hotspots in the Mediterranean Sea. Although these meadows are legally protected and included in numerous marine protected areas (MPAs), they are nevertheless threatened by direct and indirect impacts of multiple anthropogenic activities (Giakoumi et al. 2015).

In *P. oceanica* meadows, amphipods are one of the dominant groups of vagile invertebrates, forming an abundant and diverse taxocenosis (Gambi et al. 1992). They mostly feed on seagrass epiphytes with species-specific dietary preferences (Michel et al. 2015b). Through their feeding activity, they act as ecosystem engineers, as they exert selective top-down control on epiphytic assemblages and modulate nutrient availability for their seagrass host (Michel et al. 2015a). Overall, amphipod crustaceans can be considered key ecological components of the complex, pivotal, yet critically endangered Neptune grass ecosystems. Despite their ecological importance, quantitative and widely available data about amphipod fauna of Mediterranean Neptune grass meadows remain scarce. This is especially true for amphipod assemblages from meadows situated along the coasts of Mediterranean islands, whose structure has recently been showed to differ from their mainland counterparts (Bellisario et al. 2015). In this context, the aim of the AxIOM dataset is to make data collected in the framework of ecological studies freely available on the Global Biodiversity Information Facility (GBIF) portal.

## General description

### Purpose

AxIOM is a sample-based dataset (n = 187 samples) documenting occurrences of amphipod crustaceans associated to *Posidonia oceanica* seagrass meadows from Mediterranean Islands (Corsica, Sardinia). In total, it contains 1775 records, documenting occurrence and abundance of 10720 amphipod specimens belonging to 72 species spanning 29 families. Samples were collected over different periods 3 consecutive years, both during the day and during the night. A nested hierarchical sampling design was set up, and multiple sampling methods were combined to ensure a holistic view of the taxocenosis. The dataset package is composed of two data files: one describing sampling events, and the other reporting occurrence data of amphipod crustaceans.

## Project description

### Title

Multidisciplinary study of trophic diversity and functional role of amphipod crustaceans associated to Posidonia oceanica meadows AND Multiscale variability of amphipod assemblages in Posidonia oceanica meadows: A comparison between different protection levels

### Personnel

Loïc N. Michel, Nicolas Sturaro and Gilles Lepoint

### Design description

The AxIOM dataset was generated during two doctoral research programmes that took place at University of Liège, Belgium. The first one focused on ecology of amphipod crustaceans from *Posidonia oceanica* meadows, on their place in the food web and on their role in the ecosystem (Michel 2011). The second one focused on the multiscale variability patterns of amphipod assemblages associated to *P. oceanica* meadows, and their potential responses among different protection levels (Sturaro 2012).

### Funding

Belgian Fund for Scientific Research (F.R.S.-FNRS) research fellow grant nr. FC74734; Belgian Fund for Research in Industry and Agriculture (FRIA) doctoral grant.

## Sampling methods

### Study extent

AxIOM contains 187 sampling events, spanning 3 consecutive years. Sampling took place in two regions: Corsica (Calvi Bay) and Sardinia (Tavolara-Punta Coda Cavallo Marine Protected Area, TMPA). Samples were taken during different periods of the year (November, March, June, July, August) to acknowledge seasonal variation of communities (Gambi et al. 1992). Since amphipod assemblages from *Posidonia oceanica* meadows also exhibit diel variations (Sánchez-Jerez et al. 1999), samples were taken during both day and night. To ensure efficient and representative sampling of the amphipod taxocenosis, 4 complementary methods were used: hand-towed net, litter collection, air-lift and 2 slightly different types of light traps. In both investigated regions, a nested hierarchical sampling design was setup to fully capture the spatial variability of amphipod community structure over multiple scales spanning four orders of magnitude (1 to 1000 metres; Sturaro et al. 2015). Sampling stations encompass various levels of environmental protection and anthropogenic pressure, including integral reserve (TMPA zone A), partial reserve (TMPA zone B), general reserve (TMPA zone C), unprotected pristine zone (Calvi Bay) and heavily impacted zone (Gulf of Olbia).

### Sampling description

Most samples were taken following a nested hierarchical sampling design that focused on variability at 4 spatial scales, ranging from 1 to 1000 metres. In each sampling region (Corsica and Sardinia), zones separated by > 1000 m were chosen. In each zone, 2 sites (separated by ~ 100 m) were picked. Inside each site, 2 or 4 sectors (separated by ~10 m) were randomly selected within each site. Each sector was delimited by a permanent frame circumscribing an area of 9 m^2^. Depending on the method used, sampling events either covered a full sector or were taken randomly inside a sector and separated by ~1 m were collected. Details of the sampling design are given in Sturaro et al. (2015), Sturaro et al. (2014) Levels of this design are documented in the "event.txt" file of the dataset using matching hierarchized parent event IDs.

All sampling was performed by SCUBA diving at depths ranging from 10.4 to 15 metres. Detailed methodology for the hand-towed net (labelled "Net" in the "samplingProtocol" column of the "event.txt" file of the dataset), the air-lift ("Airlift") and the first type of light traps ("Trap1") can be found in Michel et al. (2010).

Litter collection ("Litter") consisted in hand-picking of litter fragments. A 25 x 40 cm quadrate was randomly thrown in the meadow, to estimate sampling area, and all litter present among this meadow patch was handpicked by fistfuls, and quickly placed in a container. By doing so, vagile organisms associated to litter fragments were also collected. This procedure was repeated until a standardized container of 2 litres was filled.

**Figure 1.**
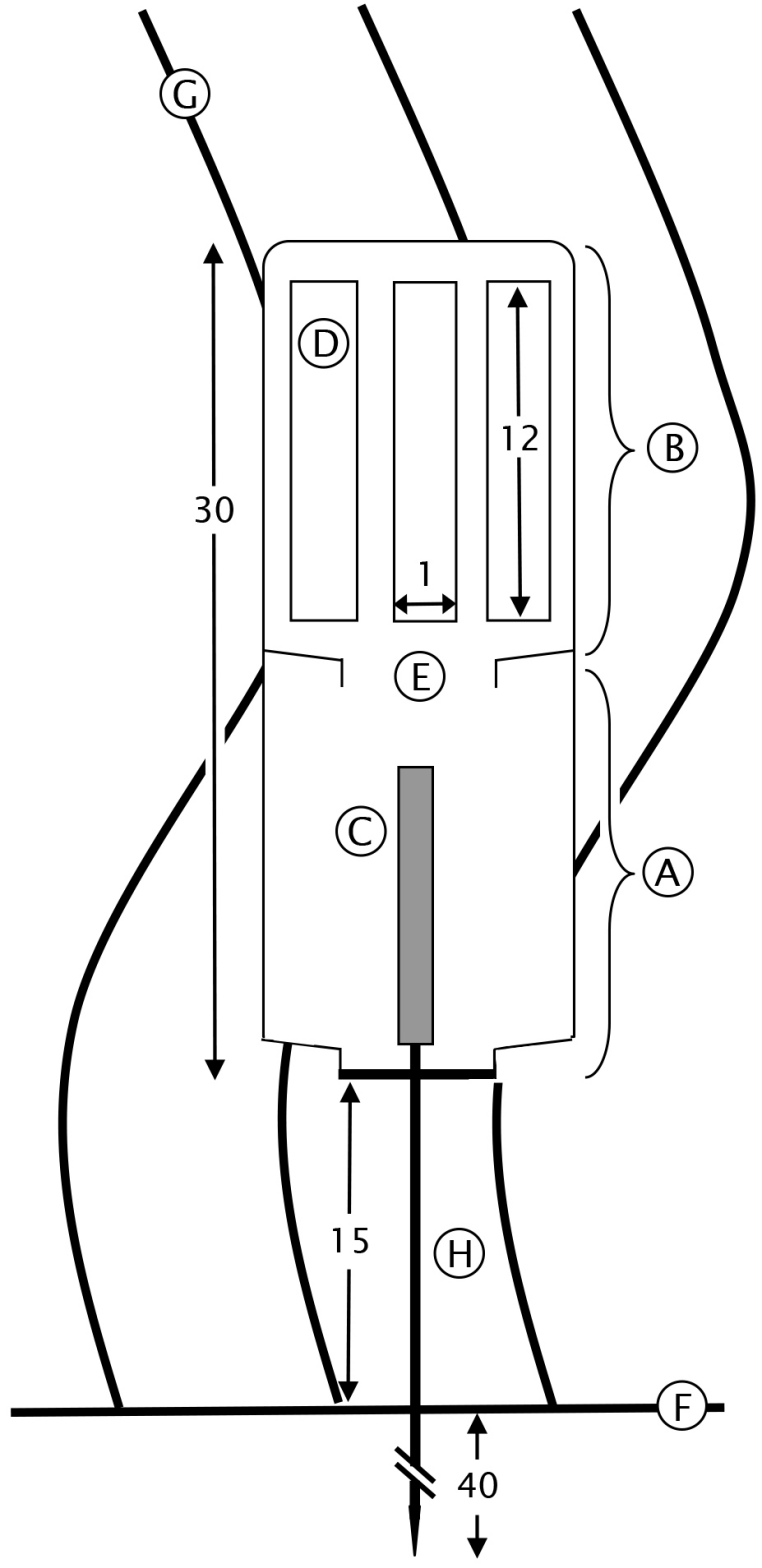
Schematic representation of the second type of light traps used in this study. All measurements are expressed in centimetres. A: bottom container, B: top container, C: diving emergency light stick, D: vertical slits, E: bottleneck, F: bottom, G: Posidonia oceanica leaves, H: metal stake.

The second type of light traps ("Trap2", Fig. 1) were made of two nested 1 litre translucent plastic containers. The top container was pierced with vertical rectangular slits (1 cm wide x 12 cm long), and was then inserted in the bottom one. Traps were anchored using metal stakes (∅: 3 mm) that were directly stuck in the matte. Each trap presented vertical rectangular slits (1 cm wide x 15 cm long) in its upper part. A diving emergency light stick was fixed in the bottom part of each trap. These sticks emit light for >12 hours, and the vagile invertebrates, attracted by the light, entered the trap through the slits. They gathered in the bottom part, the presence of a bottleneck in the middle of the trap limiting their potential escape. Traps were placed at twilight and recovered the next morning.

### Quality control

Sampling protocols were standardized to avoid biases. Amphipods were and identified using primarily the keys of the Mediterranean amphipod fauna of Bellan-Santini et al. (1982), Bellan-Santini et al. (1989), Bellan-Santini et al. (1993), Bellan-Santini et al. (1998) and the interactive key of Myers et al. (2001). In some cases, more recent diagnoses and redescriptions of species were also used. This was notably the case for the genera *Apherusa* (Krapp-Schickel and Sorbe 2006) and Caprella (Guerra-García and Takeuchi 2002, Krapp-Schickel and Takeuchi 2005, Krapp-Schickel and Vader 1998, Krapp et al. 2006). After identification, specimens were randomly selected to be re-examined by either first or last author in order to check identification accuracy. Species names were matched against the authoritative, expert-driven World Register of Marine Species (WoRMS).

### Step description

After collection, all samples were sieved on 400 µm nylon mesh to eliminate sediment and fine particulate organic matter. They were subsequently fixed for >24 hours in a formaldehyde solution (4% in 0.22 µm-filtered seawater). Samples were then sorted to isolate amphipods and transfer them to a preservation solution consisting of 70% ethanol in distilled water to which 1% glycerine was added to prevent evaporation. After identification, specimens were stored in this preservation solution in airtight vials.

## Geographic coverage

### Description

AxIOM contains samples taken in *Posidonia oceanica* seagrass meadows from Mediterranean Islands. Two regions were investigated: Corsica (Calvi Bay) and Sardinia (Tavolara-Punta Coda Cavallo marine protected area).

Calvi Bay lies in the Ligurian Sea (western Mediterranean), on the north-western coast of Corsica (France; 42°35'N, 8°45'E). It is bound by Punta Revellata Cape in the West, and by Punta Spanu Cape in the East. Temperature of water is typically minimal in February (12°C) and maximal in August (26°), with a notable vertical thermal stratification from May to September. Salinity of the water of Calvi Bay is around 38 and shows no major seasonal variation. Calvi Bay is an oligotrophic area and shows low inorganic nutrient and particulate organic matter concentrations (Lepoint et al. 2004).

In Calvi Bay, *Posidonia oceanica* meadows cover 4.94 km^2,^ *i.e.* about 50% of the area of the bay. They are found at depths ranging from 3 to 38 m. Meadows mostly grow on soft bottoms and show, in most places, a continuous extension, but local erosion (“intermattes”) occurs (Abadie et al. 2015). Meadows of Calvi Bay are relatively dense, and show an important foliar biomass and production despite the oligotrophic character of the area (Gobert et al. 2003). Overall, the coastal areas surrounding the bay are weakly urbanised and the ecological status of seawater of Calvi Bay is considered as good (Gobert et al. 2009).

The Tavolara-Punta Coda Cavallo Marine Protected Area (TMPA) lies in the Thyrrenian Sea (western Mediterranean), on the north-eastern coast of Sardinia (Italy; 40°56'N, 09°44'E). TMPA covers 153.57 km^2^ and extends along 76 km of coastline. It is located south of the Gulf of Olbia, a heavily urbanized area undergoing anthropogenic pressures from discrete (wastewater discharge and industrial activities) and diffuse (ships and coastal tourism) sources. It comprises the islands of Tavolara, Molara and Molarotto. It was established in 1997, although enforcement of protection effectively began in 2003-2004. Three zones featuring different protection regimes have been defined.

Zone A (5.29 km^2^) is an integral reserve and no-take/no-access zones. Access of zone A is restricted to scientists, reserve staff and police authorities. Zone B (31.13 km^2^) is a partial reserve where access is permitted, but only professional fishermen inhabiting the nearby coastal villages are allowed to fish. Zone C (117.15 km^2^) is a general reserve where access as well as professional and recreational fishing are allowed under restricted conditions defined by the MPA management consortium.

In TMPA, temperature of water is nearly the same as Calvi Bay, with variation between 14°C and 26°C. Salinity is around 38 and is constant the whole year. *P. oceanica* meadows cover a total surface of 4415 Ha and are found at depths ranging from 0.5 to 41 m (Tavolara-Punta Coda Cavallo Marine Protected Area management consortium pers. comm.). At sampling depth, shoot density, leaf and epiphyte biomasses do not show differences among protection levels (Sturaro et al. 2014).

### Coordinates

40.859253 and 42.579722 Latitude; 8.725000 and 9.777583 Longitude.

## Taxonomic coverage

### Description

This dataset comprises 72 amphipod species (including 2 subspecies of *Caprella acanthifera*) belonging to 51 genera and 29 families.

### Taxa included

**Table:** 

Rank	Scientific Name	Common Name
kingdom	Animalia	Animals
phylum	Arthropoda	Arthropods
subphylum	Crustacea	Crustaceans
superclass	Multicrustacea	
class	Malacostraca	
subclass	Eumalacostraca	
superorder	Peracarida	
order	Amphipoda	Amphipods
suborder	Gammaridea	
suborder	Senticaudata	
infraorder	Gammarida	
infraorder	Hadziida	
infraorder	Talitrida	
superfamily	Aoroidea	
superfamily	Caprelloidea	
superfamily	Corophioidea	
superfamily	Gammaroidea	
superfamily	Hadzioidea	
superfamily	Liljeborgioidea	
superfamily	Photoidea	
superfamily	Talitroidea	
family	Ampeliscidae	
family	Amphilochidae	
family	Ampithoidae	
family	Aoridae	
family	Atylidae	
family	Calliopiidae	
family	Caprellidae	
family	Corophiidae	
family	Cyproideidae	
family	Dexaminidae	
family	Gammaridae	
family	Hyalidae	
family	Iphimediidae	
family	Ischyroceridae	
family	Leucothoidae	
family	Liljeborgiidae	
family	Lysianassidae	
family	Maeridae	
family	Megaluropidae	
family	Nuuanuidae	
family	Oedicerotidae	
family	Opisidae	
family	Photidae	
family	Phoxocephalidae	
family	Podoceridae	
family	Pontogeneiidae	
family	Stenothoidae	
family	Uristidae	
family	Urothoidae	
genus	* Ampelisca *	
genus	* Amphilochus *	
genus	* Ampithoe *	
genus	* Aora *	
genus	* Apherusa *	
genus	* Apocorophium *	
genus	* Apolochus *	
genus	* Atylus *	
genus	* Caprella *	
genus	* Cymadusa *	
genus	* Deflexilodes *	
genus	* Dexamine *	
genus	* Ericthonius *	
genus	* Eusiroides *	
genus	* Gammarella *	
genus	* Gammaropsis *	
genus	* Gammarus *	
genus	* Gitana *	
genus	* Guernea *	
genus	* Harpinia *	
genus	* Hippomedon *	
genus	* Hyale *	
genus	* Iphimedia *	
genus	* Ischyrocerus *	
genus	* Jassa *	
genus	* Lembos *	
genus	* Leptocheirus *	
genus	* Leucothoe *	
genus	* Liljeborgia *	
genus	* Lysianassa *	
genus	* Lysianassina *	
genus	* Maera *	
genus	* Megaluropus *	
genus	* Metaphoxus *	
genus	* Microdeutopus *	
genus	* Microjassa *	
genus	* Nannonyx *	
genus	* Normanion *	
genus	* Nototropis *	
genus	* Orchomene *	
genus	* Peltocoxa *	
genus	* Perioculodes *	
genus	* Phtisica *	
genus	* Pseudolirius *	
genus	* Pseudoprotella *	
genus	* Siphonoecetes *	
genus	* Stenothoe *	
genus	* Synchelidium *	
genus	* Tmetonyx *	
genus	* Tritaeta *	
genus	* Urothoe *	
species	*Ampelisca diadema* (Costa, 1853)	
species	*Ampelisca rubella* A. Costa, 1864	
species	*Amphilochus manudens* Bate, 1862	
species	*Ampithoe helleri* Karaman, 1975	
species	*Ampithoe ramondi* Audouin, 1826	
species	*Aora gracilis* (Bate, 1857)	
species	*Aora spinicornis* Afonso, 1976	
species	*Apherusa chiereghinii* Giordani-Soika, 1949	
species	*Apocorophium acutum* (Chevreux, 1908)	
species	*Apolochus neapolitanus* (Della Valle, 1893)	
species	*Atylus massiliensis* Bellan-Santini, 1975	
species	*Atylus vedlomensis* (Bate & Westwood, 1862)	
species	*Caprella acanthifera* Leach, 1814	
species	*Caprella equilibra* Say, 1818	
species	*Caprella tavolarensis* Sturaro & Guerra-García, 2012	
species	*Cymadusa crassicornis* (Costa, 1853)	
species	*Deflexilodes griseus* (Della Valle, 1893)	
species	*Dexamine spiniventris* (Costa, 1853)	
species	*Dexamine spinosa* (Montagu, 1813)	
species	*Ericthonius punctatus* (Bate, 1857)	
species	*Eusiroides dellavallei* Chevreux, 1899	
species	*Gammarella fucicola* (Leach, 1814)	
species	*Gammaropsis dentata* Chevreux, 1900	
species	*Gammaropsis maculata* (Johnston, 1828)	
species	*Gammaropsis palmata* (Stebbing & Robertson, 1891)	
species	*Gammarus aequicauda* (Martynov, 1931)	
species	*Gammarus crinicornis* Stock, 1966	
species	*Gammarus insensibilis* Stock, 1966	
species	*Gitana sarsi* Boeck, 1871	
species	Guernea (Guernea) coalita (Norman, 1868)	
species	*Harpinia zavodniki* Karaman, 1987	
species	*Hippomedon massiliensis* Bellan-Santini, 1965	
species	*Hippomedon oculatus* Chevreux & Fage, 1925	
species	*Hyale camptonyx* (Heller, 1866)	
species	*Hyale schmidti* (Heller, 1866)	
species	*Iphimedia minuta* G.O. Sars, 1882, 1883	
species	*Ischyrocerus inexpectatus* Ruffo, 1959	
species	*Jassa ocia* (Bate, 1862)	
species	*Lembos websteri* Bate, 1857	
species	*Leptocheirus guttatus* (Grube, 1864)	
species	*Leptocheirus pectinatus* (Norman, 1869)	
species	*Leucothoe spinicarpa* (Abildgaard, 1789)	
species	*Liljeborgia dellavallei* Stebbing, 1906	
species	*Lysianassa costae* (Milne Edwards, 1830)	
species	*Lysianassa pilicornis* (Heller, 1866)	
species	*Lysianassina longicornis* (Lucas, 1846)	
species	*Maera grossimana* (Montagu, 1808)	
species	*Megaluropus massiliensis* Ledoyer, 1976	
species	*Metaphoxus simplex* (Bate, 1857)	
species	*Microdeutopus anomalus* (Rathke, 1843)	
species	*Microdeutopus similis* Myers, 1977	
species	*Microjassa cumbrensis* (Stebbing & Robertson, 1891)	
species	*Nannonyx propinquus* Chevreux, 1911	
species	*Normanion chevreuxi* Diviacco & Vader, 1988	
species	*Nototropis guttatus* Costa, 1853	
species	*Orchomene humilis* (Costa, 1853)	
species	*Orchomene similis* (Chevreux, 1912)	
species	*Peltocoxa gibbosa* (Schiecke, 1977)	
species	*Peltocoxa marioni* Catta, 1875	
species	*Perioculodes aequimanus* (Korssman, 1880)	
species	*Phtisica marina* Slabber, 1769	
species	*Podocerus variegatus* Leach, 1814	
species	*Pseudolirius kroyeri* (Haller, 1897)	
species	*Pseudoprotella phasma* (Montagu, 1804)	
species	Siphonoecetes (Centraloecetes) dellavallei Stebbing, 1899	
species	*Stenothoe cavimana* Chevreux, 1908	
species	*Stenothoe eduardi* Krapp-Schickel, 1975	
species	*Stenothoe monoculoides* (Montagu, 1815)	
species	*Synchelidium haplocheles* (Grube, 1864)	
species	*Synchelidium longidigitatum* Ruffo, 1947	
species	*Tmetonyx nardonis* (Heller, 1866)	
species	*Tritaeta gibbosa* (Bate, 1862)	
species	*Urothoe elegans* (Bate, 1857)	
subspecies	*Caprella acanthifera acanthifera* Leach, 1814	
subspecies	*Caprella acanthifera discrepans* Mayer, 1890	

## Temporal coverage

**Data range:** 2006 11 15 – 2008 8 19.

## Collection data

### Collection name

AxIOM

### Collection identifier

ULGOceano001

### Specimen preservation method

Ethanol 70% in distilled water + 1% glycerin

## Usage rights

### Use license

Other

### IP rights notes

Creative Commons CC BY

## Data resources

### Data package title

AxIOM: Amphipod crustaceans from insular Posidonia oceanica seagrass meadows

### Resource link

http://ipt.biodiversity.be/resource?r=axiom

### Number of data sets

1

### Data set 1.

#### Data set name

AxIOM: Amphipod crustaceans from insular Posidonia oceanica seagrass meadows

#### Data format

Darwin Core﻿

#### Number of columns

30

#### Download URL

http://ipt.biodiversity.be/resource?r=axiom, www.gbif.org/dataset/b146a93c-657b-4768-aa51-9cabe3dac808

#### Description

The dataset is composed of two files. The first one is named "event.txt". It gathers data about sampling events (n=187). "Event.txt" has 282 rows and 18 columns. The second file is named "occurrence.txt". It contains occurrence records (n=1775) of amphipod specimens. "Occurrence.txt" has 1776 rows and 13 columns. For more information about Darwin Core terms, please refer to http://tdwg.github.io/dwc/terms/. AxIOM has the Global Biodiversity Information Facility Universally Unique Identifier (GBIF UUID) b146a93c-657b-4768-aa51-9cabe3dac808.

**Data set 1.** 

Column label	Column description
event.txt:eventID﻿	Identification code of the sampling event (primary key).
event.txt:parentEventID	Identification code of broader, parent events that group several sampling events. To reflect the nested hierarchical sampling design (cf. "Sampling description" section of this manuscript), parent events ID were built using up to five groups of character linked, in order, to the sampling region (Corsica or Sardinia), zone, site, sector, and sampling year.
event.txt:samplingProtocol	Name of the method used to obtain the sample (cf. "Sampling description" section of this manuscript).
event.txt:sampleSizeValue	Numerical value of the sampling area.
event.txt:sampleSizeUnit	Unit used to express the sampling area (square meters).
event.txt:samplingEffort	Brief description of the amount of effort expended to obtain the sample.
event.txt:eventDate	Sampling event date. Light trap samples are taken over a full night, and therefore have two consecutive values. Precise dates were not recorded for the air-lift samples, and a date range spanning the whole sampling campaign is given instead.
event.txt:eventTime	Time interval in which the sample was taken.
event.txt:eventRemarks	For sampling events: the period of the day in which the sample was taken (day or night). For parent events: the concerned level of the nested hierarchical sampling design (cf. "Sampling description" section of this manuscript).
event.txt:waterBody	Name of the marine area in which the sample was taken.
event.txt:island	Name of the island in which the sample was taken.
event.txt:countryCode	ISO 3166-1-alpha-2 code of the country in which the sample was taken.
event.txt:minimumDepthInMeters	Minimum sampling depth, in meters.
event.txt:maximumDepthInMeters	Maximum sampling depth, in meters.
event.txt:decimalLatitude	Geographic latitude, in decimal degrees.
event.txt:decimalLongitude	Geographic longitude, in decimal degrees.
event.txt:geodeticDatum	Geodetic datum on which the geographic coordinates given in "decimalLatitude" and "decimalLongitude" are based (WGS84).
occurence.txt:basisOfRecord	Nature of the occurrence record (preserved specimen)
occurence.txt:occurrenceID	Globally unique and persistent identification number of the occurrence
occurence.txt:recordNumber	Identification number given to the occurrence at the time of record
occurence.txt:individualCount	Number of sampled specimen(s)
occurence.txt:sex	Sex of sampled specimen(s)
occurence.txt:lifeStage	Life stage of sampled specimen(s)
occurence.txt:eventID	Identification code of the sampling event (foreign key).
occurence.txt:scientificName	Binomial scientific name of the occurrence
occurence.txt:family	Family of the occurrence
occurence.txt:genus	Genus of the occurrence
occurence.txt:specificEpithet	Species epithet of the occurrence
occurence.txt:infraspecificEpithet	occurence.txt:Subspecies epithet of the occurrence
occurence.txt:taxonRank	Lowest taxonomical level to wh﻿ich the specimen(s) could be identified
